# Impact of an Organizational Climate for Evidence-Based Practice on Evidence-Based Practice Behaviour among Nurses: Mediating Effects of Competence, Work Control, and Intention for Evidence-Based Practice Implementation

**DOI:** 10.1155/2024/5972218

**Published:** 2024-07-09

**Authors:** Miao Huo, Haihong Qin, Xiaohua Zhou, Jinghua Li, Bolun Zhao, Ye Li

**Affiliations:** ^1^ School of Nursing Dalian University, Dalian 116622, China; ^2^ School of Public Health Jilin University, Changchun 130021, China; ^3^ School of Nursing Dalian Medical University, Dalian 116044, China; ^4^ Emergency Department Affiliated Zhongshan Hospital of Dalian University, Dalian 116001, China

## Abstract

**Background:**

Despite the emphasis on the importance of implementing evidence-based practices, nurses did not adopt this approach as a standard. For those who have attempted to implement evidence-based practice in health care settings, the behaviour is rarely simple or straightforward. Therefore, exploring the mechanism that motivates nurses' evidence-based practice behaviour is essential to promote this practice.

**Aims:**

The aim of this study was to investigate the effect of the organizational climate for evidence-based practice on evidence-based practice behaviour among nurses through the mediating role of evidence-based practice competence, work control, and the intention to implement evidence-based practice.

**Methods:**

This study consisted of a cross-sectional design and convenience sampling to recruit 641 nurses employed in 6 hospitals in China. Five self-report instruments were used to collect the data. A structural equation model was adopted to verify the research hypotheses. IBM SPSS 26.0 and AMOS 24.0 were used for statistical analysis of the data.

**Results:**

The organizational climate for evidence-based practice was significantly and positively related to the nurses' evidence-based practice behaviour (*p* < 0.01). Direct effects accounted for 45.93% of the total effect. Evidence-based practice competence, work control, and the intention to implement evidence-based practice partially mediated the association between the organizational climate and evidence-based practice behaviour. The indirect effect accounted for 54.07% of the total effect.

**Conclusion:**

The organizational climate for evidence-based practice is critical for predicting and enhancing evidence-based practice behaviour. Evidence-based practice competence, work control, and the intention to implement evidence-based practice are intervening mechanisms that explain how the organizational climate promotes evidence-based practice behaviour. *Implications for Nursing Management*. Nursing managers should be aware of the interaction of individual and organizational factors that influence evidence-based practice behaviours among nurses. Administrators should improve the organizational climate by providing nurses with cultural and team support, mentoring, training projects, resource provisions, and more autonomy and authority at work, which are beneficial to the nurses' evidence-based practice competence, work control, and intentions to adopt evidence-based practices.

## 1. Introduction

Evidence-based practice is conceptualized as “clinical decision-making that considers the best available evidence, the context in which the care is delivered, client preference, and the professional judgement of the health professional” [1]. The implementation of evidence-based practices can provide high-quality health care, reduce the incidence of complications, and decrease health care costs and resource use [2, 3]. However, putting evidence into practice seems complex, and the implementation of evidence-based practice continues to remain low in most health care systems [4, 5]. A survey revealed that organizational climate has the greatest influence on the implementation of evidence-based practices [6], which is defined as “reflect employees' overall assessment of their work environment” [7]. Considering that the behaviour is rarely simple or straightforward, Li et al. suggested that further research should focus on how organizational features influence implementation effectiveness [8]. The facilitation of evidence-based practice in nursing needs the organization and the individual levels [9]. Therefore, the main aim of this paper is to study the effects of the organizational climate on evidence-based practice behaviour through the mediating effect of individual factors among nurses.

For individuals, the intention to adopt evidence-based practices has been recognized as a strong precursor to subsequent utilization [10]. Consistent with the arguments in the literature, the factors that influence nurses' willingness to engage in evidence-based practices are worthy of investigation. Evidence-based practice competence was emphasized as a significant predictor of implementation [11, 12]. Nurses also experience considerable challenges in implementing evidence-based practices due to a lack of confidence in critical appraisal skills and a low level of knowledge [13–15]. It has been demonstrated that the organizational climate, such as evidence-based practice mentoring, training, and supportive culture, is associated with competence and intention of evidence-based practices [16]. The precise mechanisms and extent to which the organizational climate influences evidence-based practice behaviours through competence and intention remain unclear. More investigations that combine these factors within a single study are needed so that we can better understand their relationships.

However, the implementation of evidence-based practices continues to remain low in China, even when nurses hold strong beliefs and knowledge about evidence-based practices [17]. One possible explanation for this inconsistency may be that individuals may possess knowledge and skills but struggle to apply them flexibly in a changing environment [18]. It has been argued that control practices can also influence evidence-based practices in a dynamic work environment [19, 20]. The perception of work control results from an interaction between the individual and the work environment [21], which is defined as “a composite of decision authority (e.g., freedom to make decisions) and skill discretion (e.g., opportunity to learn new things and develop new capabilities)” [22]. According to the literature review, nurses have a low sense of work control regarding evidence-based practice, including time constraints, work stress, inadequate resources and infrastructure, and a lack of authority and autonomy to make practice changes [10, 23, 24]. All of these factors are related to the organizational climate. The interactions among the organizational climate, work control, and the intention to engage in evidence-based practice have not been clearly explored. More investigations that combine these factors within a single study are needed so that we can better understand their relationships.

Most previous studies have focused on the impact of various factors on evidence-based practice behaviour among nurses [25], and the interactions among various factors relating to this practice have not been clearly explored. This study was based on SOR (stimuli-organism-response) theory. This theory suggests that when an individual encounters a particular stimulus (S), the psychological cognition, emotional state, and ability of the individual organism (O) will change, and thus, a response (R) in the form of a behaviour will occur [26]. Therefore, the “SOR” model allows researchers to integrate various factors into systematic theory building. This study was aimed at determining how the organizational climate for evidence-based practice (S) stimulates the occurrence of evidence-based practice behaviour (R) through the mediating effect of individual factors (O), namely, evidence-based practice competence, work control, and the intention to implement evidence-based practice. For nursing practice, these results have the potential to provide nurse leaders with theory-based evidence to support strategies toward encouraging evidence-based practice behaviour among nurses and thereby improve other outcomes for nurses, patients, and health care organizations.

## 2. Literature Review and Hypotheses

**Figure 1:**
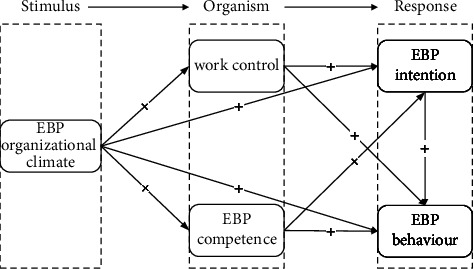
The hypothesized study model. This model presents hypothetical relationships among the variables. *Note.* “+” = positive correlation; EBP, evidence-based practice.

The aim of this study was to investigate the effect of the organizational climate for evidence-based practice on evidence-based practice behaviour among nurses through the mediating role of evidence-based practice competence, work control, and the intention to implement evidence-based practice. The hypothesized model for this study is presented in Figure 1.

### 2.1. Organizational Climate for Evidence-Based Practice and Evidence-Based Practice Behaviour

“Organizational climate” refers to “the collective understanding that organizational members attribute to the events, policies, practices, and procedures they encounter, along with the behaviors they observe being rewarded, supported, and expected” [27]. It primarily includes seven core dimensions: organizational philosophy, environmental atmosphere, work style, teamwork, leader support, training, and resource provision [28]. Empirical studies have shown that it represents a single, overarching factor that captures employee evaluations of how the work environment impacts their personal well-being [29]. According to the previous research study, the barriers from organizational factors to the implementation of evidence-based practice include inadequate resources and infrastructures, lack of leadership support and evidence-based practice mentors, a weak culture of evidence-based practice, and less collaboration between academics and clinics [10, 20, 30]. Most of the literature focuses on a single aspect of the organizational climate for evidence-based practice. Therefore, this study integrates existing organizational factors of evidence-based practice into the concept of organizational climate for evidence-based practice, which represents an organization's larger social context. The first aim of this study was to examine whether an organizational climate for evidence-based practice that integrates multiple organizational factors could promote nurses' evidence-based practice behaviour.

**Hypothesis 1 .**

The organizational climate for evidence-based practice is positively and directly related to nurses' evidence-based practice behaviour.

### 2.2. Evidence-Based Practice Competence and the Intention to Implement Evidence-Based Practice as Mediators

In addition, we attempt to explore the mechanisms through which an organizational climate for evidence-based practice enhances evidence-based practice behaviour among nurses. Evidence-based practice competence and the intention to adopt evidence-based practice have been recognized as strong precursors to their subsequent utilization and actual practice [12, 25]. For individuals to implement evidence-based practice effectively, nurses need to be motivated in which they have a desire to seek out the best information that serves the needs of their patients, and they need to be competent in which they must have the necessary knowledge, skills, and attitudes that can be linked to evidence-based practice [24, 31]. However, the findings indicated that the nurses did not feel prepared for evidence-based practice. A systematic review of 18,355 nurses from 21 countries revealed that nurses did not use the best evidence in practice because of insufficient evidence-based practice knowledge and skills [30]. The lack of evidence-based practice competence results in lower confidence in implementing these practices.

Notably, the competence and intention underlying evidence-based practice have been associated with the organizational climate, such as the evidence-based practice mentoring, training, and supportive culture [16]. A survey revealed that advanced practice nurses, as “opinion leaders,” significantly influence the evidence-based practices of front-line nurses [23], and the top three sources of evidence-based practices that nurses obtained were information from specialists, instructors, and senior nurses [32]. Therefore, evidence-based practice mentoring can promote the competence of nurses in successfully engaging in evidence-based practice. In addition, training was seen as important in explaining the likelihood of future implementation of evidence in clinical nursing practice [10]. The reason is that after continuing education programs of evidence-based practice in hospitals, nurses' evidence-based practice knowledge scores improved [33]. The findings of Melnyk demonstrated evidence-based practice culture as a key variable that directly affects evidence-based practice knowledge, beliefs, and competency [5]. Therefore, this study was aimed at examining whether an organizational climate for evidence-based practice could promote this behaviour among nurses through evidence-based practice competence and intention.

**Hypothesis 2 .**

Evidence-based practice competence mediates the relationship between the organizational climate and evidence-based practice behaviour.

**Hypothesis 3 .**

The intention to implement evidence-based practice mediates the relationship between the organizational climate and evidence-based practice behaviour.

**Hypothesis 4 .**

Evidence-based practice competence and the intention to implement evidence-based practice play a chain mediating role between the organizational climate and evidence-based practice behaviour.

### 2.3. Work Control and the Intention to Implement Evidence-Based Practice as Mediators

Work control is an essential work characteristic that is generally defined as “one's control over one's task, conduct, and performance or the ability to have influence over one's work and work environment to obtain a rewarding work situation, such as control of work efficiency (e.g., the method, amount, and speed for increasing efficiency), control of work resources (e.g., the perception of authority in regard to job-related information, procedures, or materials for meeting the demands of the job), and control of work environment (e.g., decoration of the work area and protection of the working environment from interference)” [34]. A sense of high work control has been demonstrated to increase work engagement and stimulate intrinsic motivation [35]. Under conditions of high work control, nurses' performance in relation to evidence-based practice is actually fostered.

According to the literature review, the barriers to implement evidence-based practice are insufficient time for involvement in this practice, inadequate resources and infrastructure, and a lack of authority and autonomy to make practice changes [10, 24]. This result suggests that nurses have a low sense of work control when implementing evidence-based practice. When nurses perceive a lack of control over their work, particularly in relation to resources and the environment, they are less inclined to engage in evidence-based practice, even if they have sufficient evidence-based practice competence. This low engagement occurs because these factors are often beyond the control of the individual [24]. The provision of significant support to nurses, including time, funding, administrative support, and mentors [19], enables nurses to exert greater control over their resources, environment, and efficiency. This control may facilitate nurses' willingness to implement evidence-based practices. Although existing studies lack enough investigation and further study about nurses' work control in evidence-based practice, there may be a relationship among work control, intention to implement evidence-based practice, and organizational climate for evidence-based practice. However, the exact underlying mechanism remains unclear. Therefore, this study examined whether an organizational climate for evidence-based practice could promote nurses' evidence-based practice behaviour through work control and intention.

**Hypothesis 5 .**

Work control mediates the relationship between the organizational climate and evidence-based practice behaviour.

**Hypothesis 6 .**

Work control and the intention to implement evidence-based practice play a chain mediating role between the organizational climate and evidence-based practice behaviour.

## 3. Methods

### 3.1. Design

This study was a descriptive, cross-sectional survey that gathered data from nurses in China in 2022.

### 3.2. Participants

The survey was conducted with a population of nurses from 6 hospitals in 6 cities in China: Beijing, Shanghai, Guangzhou, Zhengzhou, Shenyang, and Urumqi. With the help of the nursing managers, surveys were sent to the nurses using a convenient sampling method. The inclusion criteria were as follows: those who (a) had obtained the professional qualification nursing certificate from the People's Republic of China, (b) were working in the hospital during the investigation period, and (c) had at least 1 year of evidence-based practice experience. The exclusion criteria counted those who were not willing to participate or were absent during the survey.

### 3.3. Data Collection

Although the minimum sample size for structural equation model analysis is 200 [36], 380 to 760 participants would be considered to be the optimal sample size because of the 76 items included, according to the principle that the sample size is approximately 5 to 10 times the number of scale items [37]. Assuming a 20% attrition rate based on previous studies conducted in China [38], the minimum sample size was 456.

We explained the purpose and method of this study to the nursing managers of each hospital, invited them to serve as research assistants, and provided them with training on the study. After learning about the implementation of evidence-based practices in the hospital and obtaining permission, the researchers or trained research assistants distributed the questionnaires to the clinical departments that met the inclusion criteria. All the data were collected using electronic questionnaires. The purpose of this study was explained, and detailed instructions were given to the guide nurses about filling out the questionnaires. After the anonymity and confidentiality of participation were explained, the nurses were informed that they were free to refuse to participate or withdraw from participation at any time without penalty. A total of 700 questionnaires were distributed, 650 of which were completed and returned to the researchers. After being checked by 2 researchers, 9 electronic questionnaires were excluded because they had the same answers (e.g., all 4 s or all 5 s), and 641 questionnaires (91.57%) were determined to be valid.

### 3.4. Instruments

Five self-report instruments were employed in this study. Two of these are widely used in China (Work Control Scale and Evidence-Based Behaviour Scale). Two were self-developed (Intention to Implement Evidence-Based Practice Scale and Organizational Climate for Evidence-Based Practice Scale), and one was a Chinese translation of the English version (Evidence-Based Practice Competency for Practicing Registered Nurses Scale). The self-developed or translated scales were tested in two stages, with stage 1 comprising the creation (or translation) and adaptation of the scales and stage 2 evaluating the psychometric properties of the scales. These 3 instruments were initially pretested among eligible participants who were excluded from this study.

#### 3.4.1. Work Control

The 19-item Chinese version of the Work Control Scale for nurses [34] comprises three domains: control of work efficiency, control of work resources, and control of work environment. A 5-point Likert scale is used, ranging from 1 (almost not) to 5 (very much), with higher scores indicating greater levels of work control. The internal consistency reliability (0.90) and test-retest reliability (0.77) of the scale indicated good reliability [34]. Cronbach's *α* was 0.930 in this study.

#### 3.4.2. Evidence-Based Practice Behaviour

Evidence-based practice behaviour was measured using the Chinese version of the Evidence-Based Practice Questionnaire (EBPQ) [39]. It comprises 3 subscales: use of evidence-based practice, attitude towards evidence-based practice, and knowledge/skills associated with evidence-based practice. The subscale “use of evidence-based practice” was adopted to measure the implementation of evidence-based practice by Anaman-Torgbor et al. [40] and consists of 6 items. All items are scored on a 7-point Likert scale ranging from 1 (never) to 7 (always), with a higher score indicating a more positive implementation of evidence-based practice. Cronbach's *α* of the subscale was 0.84 [39]. Cronbach's *α* was 0.911 in this study.

#### 3.4.3. Evidence-Based Practice Competence

The Evidence-Based Practice Competency for Practicing Registered Nurses Scale [16] was modified by our team using the “translation-back-translation-cultural adaptation” procedure in accordance with Chinese cultural background. This scale consists of 13 items, which are rated on a 4-point Likert scale ranging from 1 (not competent) to 4 (highly competent). A higher score indicates a higher level of self-rated competence. The internal consistency was 0.98 [5]. Cronbach's *α* was 0.917 in this study.

#### 3.4.4. Intention to Implement Evidence-Based Practices

The nurses' intentions to implement evidence-based practices were measured by using a scale developed by our team in this study. This scale consists of 4 items, which are rated on a 5-point Likert scale ranging from 1 (strongly disagree) to 5 (strongly agree), with higher scores indicating greater levels of intention to implement evidence-based practice. Cronbach's *α* was 0.865 in this study.

#### 3.4.5. Organizational Climate for Evidence-Based Practice

The organizational climate for evidence-based practice was measured by using a scale developed by our team in this study. It consists of 28 items divided into 7 dimensions, including the organizational philosophy, environmental atmosphere, work style, teamwork, leader support, training, and resource provision. All the items are scored on a 5-point Likert scale ranging from 1 (strongly disagree) to 5 (strongly agree). Higher scores indicated a stronger organizational climate for evidence-based practice. The scale has good internal consistency for the overall scale (Cronbach's *α* = 0.948) and 7 dimensions (Cronbach's *α* = 0.851, 0.810, 0.831, 0.887, 0.869, 0.883, and 0.872) in this study.

### 3.5. Data Analysis

IBM SPSS 26.0 and AMOS 24.0 were used for the statistical analysis of the data. Descriptive statistics were used to quantify the collected data. The mean and standard deviation were used to describe continuous variables, and the frequency and percentage were used to describe categorical variables. The correlations among evidence-based practice competence, work control, the intention to implement evidence-based practice, the organizational climate, and evidence-based practice behaviour were analysed using Pearson correlations. Structural equation modelling was used to test the hypothesized study model. The following criteria were used to evaluate the model: *χ*^2^/df < 5, RMSEA < 0.08, GFI > 0.9, NFI > 0.9, IFI > 0.9, TLI > 0.9, and CFI > 0.9 [41]. The bootstrap method was used to iterate 5000 times to estimate the mediating effect. The confidence intervals were 95% confidence intervals and did not contain 0, which signifies statistical significance.

### 3.6. Ethical Approval

Ethical approval was not needed because no unethical behaviour was present in this study, and our study did not involve human clinical trials or animal experiments. The professionals were invited to participate voluntarily through the electronic questionnaire. They were informed about the objectives of the study, with clarification that their participation was completely anonymous and that submitting the questionnaire granted their consent for participating in the study.

## 4. Results

### 4.1. Demographic Profile of Participants

Table 1 shows that most of the nurses who completed the survey (*n* = 641) were female (97.04%), had worked in the Department of Internal Medicine (64.27%), had a bachelor's degree (79.72%), had teaching experience (52.57%), had research experience (24.80%), and had 6–15 years of clinical experience (58.97%).

### 4.2. Descriptive Statistics and Correlational Analysis of Variables

As presented in Table 2, the mean overall score of the organizational climate for evidence-based practice was 3.66 (SD = 0.57), the evidence-based practice competency score was 2.65 (SD = 0.55), the work control score was 3.48 (SD = 0.60), the intention to implement evidence-based practice score was 3.90 (SD = 0.81), and the evidence-based practice behaviour score was 4.16 (SD = 1.32). The correlation analysis results show that there were positive impacts among the independent variable (organizational climate for evidence-based practice), the dependent variable (evidence-based practice behaviour), and the mediating variables (competence, work control, and intention for evidence-based practice implementation). Tables S1–S5 show the results of the *t*-test or analysis of variance (ANOVA) for the variables above.

### 4.3. Verification of Research Hypotheses

**Figure 2:**
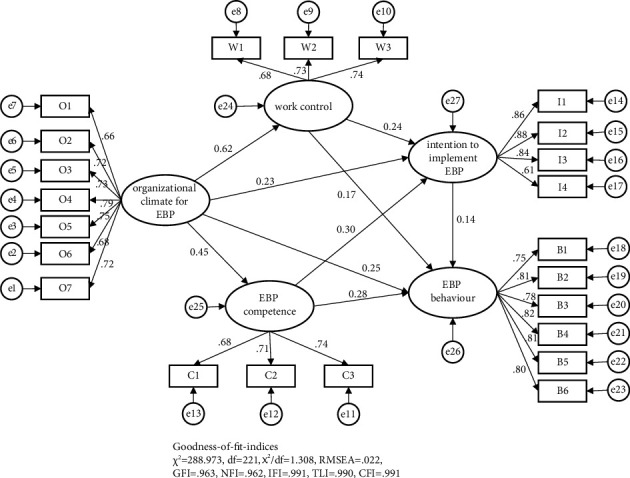
Mediation model. This model describes the paths between the variables and shows the standardized path coefficients. *Note*. EBP, evidence-based practice.

First, we assessed the measurement model, which included five latent constructs (organizational climate for evidence-based practice, evidence-based practice competence, work control, intention to implement evidence-based practice, and evidence-based practice behaviour) and 23 observational variables. Confirmatory factor analysis revealed that the model fit the data well (*χ*^2^ = 288.973, df = 221, *χ*^2^/df = 1.308, RMSEA = 0.022, GFI = 0.963, NFI = 0.962, IFI = 0.991, TLI = 0.990, and CFI = 0.991) (see Figure 2), and all the indicators were significantly loaded on the corresponding constructs.

Second, as presented in Figure 2, we verified whether the organizational climate for evidence-based practice positively affects evidence-based practice behaviour (Hypothesis 1).  Table 3 shows that the organizational climate for evidence-based practice significantly affected evidence-based practice behaviour (*β* = 0.25, *p* < 0.001).  Table 4 reveals the direct, indirect, and total effects of the final model. The results demonstrated that the 95% confidence intervals of all of the effects did not overlap with zero, which indicated that all the direct and indirect effects were significant. Direct effects accounted for 45.93% of the total effect. This result indicates that the organizational climate for evidence-based practice can predict evidence-based practice behaviour well.

Lastly, we tested a mediating effect model to verify whether evidence-based practice competence, work control, and intention to implement evidence-based practice mediate the relationship between organizational climate and evidence-based practice behaviour.  Table 3 shows that organizational climate for evidence-based practice significantly affected evidence-based practice competence (*β* = 0.45, *p* < 0.001), which in turn had a positive effect on evidence-based practice behaviour (*β* = 0.28, *p* < 0.001). The indirect effect of the organizational climate on evidence-based practice behaviour via evidence-based practice competence accounted for 22.24% of the total effect (Hypothesis 2). The organizational climate for evidence-based practice significantly affected the intention to implement evidence-based practice (*β* = 0.23, *p* < 0.001), which had a positive effect on evidence-based practice behaviour (*β* = 0.14, *p*=0.003). The indirect effect of the organizational climate on evidence-based practice behaviour via intention accounted for 5.79% of the total effect (Hypothesis 3). The organizational climate for evidence-based practice significantly affected the work control (*β* = 0.62, *p* < 0.001), which had a positive effect on evidence-based practice behaviour (*β* = 0.17, *p*=0.002). The indirect effect of the organizational climate on evidence-based practice behaviour via work control accounted for 18.99% of the total effect (Hypothesis 5). The data indicated that evidence-based practice competence, work control, and intention played a partial mediating role between the organizational climate and evidence-based practice behaviour, and the indirect effect accounted for 47.02% of the total effect. Moreover, evidence-based practice competence significantly affected the intention to implement evidence-based practice (*β* = 0.30, *p* < 0.001), and work control significantly affected the intention to implement evidence-based practice (*β* = 0.24, *p* < 0.001). The data demonstrated that competence and intention, as well as work control and intention, played a chain mediating role in the influence of the organizational climate on evidence-based practice behaviour (Hypotheses 4 and 6). The chain indirect effects accounted for 3.25% and 3.80% of the total effects, respectively.

## 5. Discussion

According to the analysis of the survey data, the organizational climate for evidence-based practice positively affected evidence-based practice behaviour. This finding was supported by a study that revealed that nurses working in departments with better organizational contextual features (organizational culture; leadership; networks and communication; resources; evaluation, monitoring, and feedback; and champions) engaged in greater use of evidence-based practice [8]. Leadership engagement, the availability of resources, and the provision of educational support, which are the three major components of organizational climate, are all key indicators of whether practice climates are conducive to evidence-based practice implementation [27]. This is because nurses will implement evidence-based practices more proactively to a greater extent when they perceive their organizational climate as more supportive. In addition, positive feedback from nursing managers can enhance nurses' sense of responsibility and accomplishment and foster the use of evidence-based practices in a virtuous circle. Thus, improving the organizational climate for evidence-based practice in the department makes great sense in promoting evidence-based practice behaviour.

In this survey, evidence-based practice competence mediated the relationship between the organizational climate and evidence-based practice behaviour, which contributed the most to the indirect effect. We can predict that professional nurses present weakness in the implementation of evidence-based practice related to the lack of evidence-based practice competence. This finding is consistent with the results of previous research [42, 43]. Research has demonstrated that nursing education and training have a positive influence on the successful achievement of evidence-based practice competence [44]. However, prior studies have typically focused on “theory-based” education, which does not meet the needs of complex, dynamic clinical environments [31]. Researchers refer to this issue as the “theory-practice gap.” Nurses face changes in the context of health care services, such as the emergence of constantly updated evidence, medical knowledge and technology, different stakeholder attitudes, and dynamic clinical resources and conditions. Therefore, we should attempt to improve evidence-based practice competence continuously, not only during the undergraduate years but also throughout the careers of health professionals. As one of the core factors during a professional career, a good organizational climate can promote evidence-based practice competence in the following ways: (a) ensuring nurses' knowledge and skills in complex clinical environments (e.g., through “practice-based” training, mentoring, teamwork, and work styles) and (b) enhancing attitudes and beliefs about evidence-based practices (e.g., through leader support, culture, and resource provision).

Furthermore, work control mediated the relationship between organizational climate and evidence-based practice behaviour, which contributed to the second indirect effect. Although evidence-based practice competence is considered the core factor of implementing evidence-based practice, some studies have found that even nurses who possess evidence-based practice competence are not necessarily able to use it flexibly in a changeable environment [18]. This lack of application is a potential result of neglecting nurses' perceptions of their environment, resource reconfiguration, and control of themselves. This study revealed that work control, which is the ability to cope with the actual working situation, may be another important individual factor affecting evidence-based practice behaviour. According to our survey, nurses generally believe that controlling resources (e.g., financial resources, policy, staffing, and infrastructure) and the environment (e.g., atmosphere and work style) are the most difficult factors, and these issues can be solved by improving the organizational climate for evidence-based practice. In addition, nursing managers need to provide nurses with more opportunities and incentives to promote evidence-based practices, such as further study, bonuses, and staff development. Managers can improve nurses' control of their work by (a) creating more opportunities for nurses to work more freely and autonomously; (b) attracting nurses to participate in management decisions, such as the formulation of department rules and regulations, performance evaluations, and salary incentive systems; and (c) assigning young and highly educated nurses with the ability to challenge and innovate work tasks to enhance their sense of accomplishment.

Lastly, the study demonstrated that the intention to implement evidence-based practice mediates the relationship between organizational climate and evidence-based practice behaviour. Moreover, evidence-based practice competence and the intention to implement evidence-based practices had a chain mediating effect on the relationship between organizational climate and evidence-based practice behaviour, and work control and intention had the same chain mediating effect. Intention is the tendency and motivation before taking action, which is seen as a strong precursor to the subsequent utilization of evidence-based practice [45]. A more recent study also identified several factors that strengthened intentions to adopt evidence-based practices, including nurses' capabilities, beliefs, attitudes, support received from nurses and other faculty members, adequate clinical and academic support, and Internet and journal access [46, 47]. Nurses are more confident and willing to use evidence-based practices, which is significantly associated with both nurse variables (evidence-based practice competence and control of work) and organizational variables (organizational climate for evidence-based practice).

## 6. Limitations

There are several limitations in our study that need to be improved upon through follow-up research. First, our study was conducted in the form of a self-report questionnaire, and the results are relatively subjective. Second, our research was only performed in some provinces in China. Therefore, the sample has some limitations. In future research, we will further expand the sample size and involved regions to make the sample more representative.

## 7. Conclusions

This study confirmed that the organizational climate for evidence-based practice is critical for predicting and enhancing evidence-based practice behaviour. Evidence-based practice competence, work control, and the intention to implement evidence-based practice are intervening mechanisms that explain how organizational climate promotes evidence-based practice behaviour.

## 8. Implication for Nursing Management

Our findings highlight the need for nurse managers to be aware that the organizational factor (the organizational climate for evidence-based practice) influences evidence-based practice behaviour through individual factors (evidence-based practice competence, work control, and intention to implement evidence-based practice). This relationship suggests that nursing managers should pay attention not only to the factors influencing evidence-based practice behaviour but also the mechanism for the interaction between these factors. Although evidence-based practice competence is the most important individual factor for evidence-based practice behaviour according to previous studies, work control should be enhanced as another important individual factor. Managers should improve the organizational climate by providing nurses with culture and team support, training projects, and resource provisions. They should also enforce more autonomy and authority at work, which are beneficial to evidence-based practice competence, work control, and the intention to adopt evidence-based practice.

## Figures and Tables

**Table 1:** Demographic profile of the participants (*n* = 641).

Characteristics	Frequency (f)	Percentage (%)
Sex		
Male	19	2.96
Female	622	97.04
Highest educational attainment		
College	108	16.85
Bachelor	511	79.72
Master	22	3.43
Doctorate	0	0.00
Working years		
≤5	157	24.49
6–10	203	31.67
11–15	175	27.30
16–20	55	8.58
≥21	51	7.96
Teaching experience		
Yes	337	52.57
No	304	47.43
Research experience		
Yes	159	24.80
No	482	75.20
Department		
Department of internal medicine	412	64.27
Department of surgery	87	13.57
Department of gynaecology	28	4.37
Department of paediatrics	28	4.37
Emergency room	7	1.09
Other departments	79	12.32

**Table 2:** Descriptive statistics and Pearson correlation of all the variables (*n* = 641).

	Cronbach's *α*	Mean (SD)	1	2	3	4	5
(1) Organizational climate for EBP	0.94	3.66 (0.57)	1				
(2) EBP competence	0.89	2.65 (0.55)	0.37^*∗∗*^	1			
(3) Work control	0.92	3.48 (0.60)	0.50^*∗∗*^	0.23^*∗∗*^	1		
(4) EBP intention	0.87	3.90 (0.81)	0.45^*∗∗*^	0.39^*∗∗*^	0.38^*∗∗*^	1	
(5) EBP behaviour	0.89	4.16 (1.32)	0.50^*∗∗*^	0.42^*∗∗*^	0.40^*∗∗*^	0.43^*∗∗*^	1

*Note*. ^*∗∗*^*P* < 0.01; EBP, evidence-based practice.

**Table 3:** Path coefficient between variables.

Path	Standardized *β*	Unstandardized B	S.E.	*t*	*P*
Organizational climate for EBP ⟶ EBP behaviour	0.25	0.55	0.12	4.49	<0.001
EBP competence ⟶ EBP behaviour	0.28	0.71	0.13	5.55	<0.001
Work control ⟶ EBP behaviour	0.17	0.44	0.15	3.05	0.002
Intention to implement EBP ⟶ EBP behaviour	0.14	0.21	0.07	2.96	0.003
Organizational climate for EBP ⟶ intention to implement EBP	0.23	0.33	0.08	3.89	<0.001
EBP competence ⟶ intention to implement EBP	0.30	0.51	0.08	6.07	<0.001
Work control ⟶ intention to implement EBP	0.24	0.42	0.10	4.21	<0.001
Organizational climate for EBP ⟶ work control	0.62	0.51	0.05	11.23	<0.001
Organizational climate for EBP ⟶ EBP competence	0.45	0.37	0.04	8.78	<0.001

*Note.* EBP, evidence-based practice.

**Table 4:** Confidence interval of mediating effect value.

Path	Effect	S.E.	95% LCI	95% UCI	Ratio (%)
Direct effect					
EBP organizational climate ⟶ EBP behaviour	0.254	0.075	0.107	0.402	45.93
Total indirect effect	0.299	0.056	0.192	0.411	54.07
EBP organizational climate ⟶ work control ⟶ EBP behaviour	0.105	0.051	0.014	0.210	18.99
EBP organizational climate ⟶ EBP competence ⟶ EBP behaviour	0.123	0.040	0.060	0.221	22.24
EBP organizational climate ⟶ intention to implement EBP ⟶ EBP behaviour	0.032	0.019	0.003	0.079	5.79
EBP organizational climate ⟶ work control ⟶ intention to implement EBP ⟶ EBP behaviour	0.021	0.011	0.004	0.050	3.80
EBP organizational climate ⟶ EBP competence ⟶ intention to implement EBP ⟶ EBP behaviour	0.018	0.009	0.003	0.042	3.25
Total effect	0.553	0.045	0.462	0.638	100.00

*Note.* EBP, evidence-based practice.

## Data Availability

The data that support the findings of this study are available upon request from the corresponding author. The data are not publicly available due to privacy or ethical restrictions.

## Supplementary Materials

Supplementary Materials Tables S1–S5 show the results of the *t*-test or analysis of variance (ANOVA) for the following variables: organizational climate, evidence-based practice competence, work control, and intention to implement evidence-based practice and evidence-based practice behaviour.
