# Supplementary material for: Impact of an Organizational Climate for Evidence-Based Practice on Evidence-Based Practice Behaviour among Nurses: Mediating Effects of Competence, Work Control, and Intention for Evidence-Based Practice Implementation
Source: J Nurs Manag. 2024 Jul 9;2024:5972218. doi: 10.1155/2024/5972218 (PMC11919118; doi:10.1155/2024/5972218)
Supplement: Supplementary Materials — Tables S1–S5 show the results of the t-test or analysis of variance (ANOVA) for the following variables: organizational climate, evidence-based practice competence, work control, and intention to implement evidence-based practice and evidence-based practice behaviour. [file 5972218.f1.docx]

**Supplementary Materials**

Table S1: Results of the t-test for variables between different genders

| Variable | Attribute | N | Mean | Std. Deviation | t | P |
| --- | --- | --- | --- | --- | --- | --- |
| Organizational Climate | Male | 19 | 3.639 | 0.789 | -0.146 | 0.884 |
|  | Female | 622 | 3.658 | 0.567 |  |  |
| EBP competence | Male | 19 | 2.530 | 0.809 | -0.944 | 0.346 |
|  | Female | 622 | 2.651 | 0.540 |  |  |
| Work Control | Male | 19 | 3.534 | 0.726 | 0.438 | 0.662 |
|  | Female | 622 | 3.473 | 0.594 |  |  |
| EBP intention | Male | 19 | 3.855 | 1.025 | -0.268 | 0.789 |
|  | Female | 622 | 3.906 | 0.805 |  |  |
| EBP behaviour | Male | 19 | 4.272 | 1.658 | 0.367 | 0.714 |
|  | Female | 622 | 4.159 | 1.309 |  |  |

Table S2: Results of the analysis of variance (ANOVA) for the variables among different highest educational attainment levels

| Variable | Attribute | N | Mean | Std. Deviation | F | P |
| --- | --- | --- | --- | --- | --- | --- |
| Organizational Climate | College | 108 | 3.530 | 0.562 | 5.165 | 0.006 |
|  | Bachelor | 511 | 3.674 | 0.570 |  |  |
|  | Master | 22 | 3.915 | 0.591 |  |  |
|  | Doctorate | 0 | 0 | 0 |  |  |
| EBP competence | College | 108 | 2.496 | 0.553 | 6.518 | 0.002 |
|  | Bachelor | 511 | 2.669 | 0.535 |  |  |
|  | Master | 22 | 2.875 | 0.709 |  |  |
|  | Doctorate | 0 | 0 | 0 |  |  |
| Work Control | College | 108 | 3.326 | 0.539 | 4.154 | 0.016 |
|  | Bachelor | 511 | 3.504 | 0.601 |  |  |
|  | Master | 22 | 3.544 | 0.700 |  |  |
|  | Doctorate | 0 | 0 | 0 |  |  |
| EBP intention | College | 108 | 3.551 | 0.789 | 14.706 | 0.000 |
|  | Bachelor | 511 | 3.962 | 0.800 |  |  |
|  | Master | 22 | 4.296 | 0.689 |  |  |
|  | Doctorate | 0 | 0 | 0 |  |  |
| EBP behaviour | College | 108 | 3.866 | 1.336 | 6.793 | 0.001 |
|  | Bachelor | 511 | 4.192 | 1.297 |  |  |
|  | Master | 22 | 4.939 | 1.404 |  |  |
|  | Doctorate | 0 | 0 | 0 |  |  |

Table S3: Results of the analysis of variance (ANOVA) for the variables among different working years

| Variable | Attribute | N | Mean | Std. Deviation | F | P |
| --- | --- | --- | --- | --- | --- | --- |
| Organizational Climate | ≤5 | 157 | 3.727 | 0.525 | 5.533 | 0.000 |
|  | 6-10 | 203 | 3.674 | 0.551 |  |  |
|  | 11-15 | 175 | 3.571 | 0.578 |  |  |
|  | 16-20 | 55 | 3.459 | 0.664 |  |  |
|  | >20 | 51 | 3.891 | 0.585 |  |  |
| EBP competence | ≤5 | 157 | 2.657 | 0.502 | 0.508 | 0.730 |
|  | 6-10 | 203 | 2.637 | 0.550 |  |  |
|  | 11-15 | 175 | 2.663 | 0.527 |  |  |
|  | 16-20 | 55 | 2.561 | 0.677 |  |  |
|  | >20 | 51 | 2.696 | 0.612 |  |  |
| Work Control | ≤5 | 157 | 3.583 | 0.624 | 3.273 | 0.011 |
|  | 6-10 | 203 | 3.455 | 0.616 |  |  |
|  | 11-15 | 175 | 3.394 | 0.485 |  |  |
|  | 16-20 | 55 | 3.373 | 0.713 |  |  |
|  | >20 | 51 | 3.613 | 0.605 |  |  |
| EBP intention | ≤5 | 157 | 3.998 | 0.796 | 4.647 | 0.001 |
|  | 6-10 | 203 | 3.930 | 0.814 |  |  |
|  | 11-15 | 175 | 3.810 | 0.810 |  |  |
|  | 16-20 | 55 | 3.595 | 0.751 |  |  |
|  | >20 | 51 | 4.172 | 0.804 |  |  |
| EBP behaviour | ≤5 | 157 | 4.320 | 1.280 | 3.086 | 0.016 |
|  | 6-10 | 203 | 4.132 | 1.367 |  |  |
|  | 11-15 | 175 | 4.005 | 1.194 |  |  |
|  | 16-20 | 55 | 3.924 | 1.479 |  |  |
|  | >20 | 51 | 4.598 | 1.367 |  |  |

Table S4: Results of the t-test for the variables between different teaching experiences

| Variable | Attribute | N | Mean | Std. Deviation | t | P |
| --- | --- | --- | --- | --- | --- | --- |
| Organizational Climate | Yes | 337 | 3.695 | 0.586 | 1.752 | 0.080 |
|  | No | 304 | 3.616 | 0.557 |  |  |
| EBP competence | Yes | 337 | 2.683 | 0.562 | 1.752 | 0.080 |
|  | No | 304 | 2.607 | 0.533 |  |  |
| Work Control | Yes | 337 | 3.498 | 0.563 | 1.008 | 0.314 |
|  | No | 304 | 3.450 | 0.635 |  |  |
| EBP intention | Yes | 337 | 3.995 | 0.802 | 2.987 | 0.003 |
|  | No | 304 | 3.804 | 0.812 |  |  |
| EBP behaviour | Yes | 337 | 4.272 | 1.334 | 2.219 | 0.027 |
|  | No | 304 | 4.041 | 1.294 |  |  |

Table S5: Results of the t-test for the variables between different research experiences

| Variable | Attribute | N | Mean | Std. Deviation | t | P |
| --- | --- | --- | --- | --- | --- | --- |
| Organizational Climate | Yes | 159 | 3.729 | 0.589 | 1.817 | 0.070 |
|  | No | 482 | 3.634 | 0.567 |  |  |
| EBP competence | Yes | 159 | 2.738 | 0.621 | 2.429 | 0.015 |
|  | No | 482 | 2.617 | 0.521 |  |  |
| Work Control | Yes | 159 | 3.586 | 0.607 | 2.698 | 0.007 |
|  | No | 482 | 3.439 | 0.591 |  |  |
| EBP intention | Yes | 159 | 4.083 | 0.785 | 3.229 | 0.001 |
|  | No | 482 | 3.845 | 0.812 |  |  |
| EBP behaviour | Yes | 159 | 4.592 | 1.327 | 4.818 | 0.000 |
|  | No | 482 | 4.021 | 1.287 |  |  |
